# Finding a Needle in a Haystack: Distinguishing Mexican Maize Landraces Using a Small Number of SNPs

**DOI:** 10.3389/fgene.2017.00045

**Published:** 2017-04-18

**Authors:** Jose L. Caldu-Primo, Alicia Mastretta-Yanes, Ana Wegier, Daniel Piñero

**Affiliations:** ^1^Laboratorio de Genética de la Conservación, Jardín Botánico, Instituto de Biología, Universidad Nacional Autónoma de México, Ciudad UniversitariaCoyoacán, Mexico; ^2^CONACYT/CONABIO, Comisión Nacional para el Conocimiento y Uso de la BiodiversidadTlalpan, Mexico; ^3^Departamento de Ecología Evolutiva, Instituto de Ecología, Universidad Nacional Autónoma de México, Ciudad UniversitariaCoyoacán, Mexico

**Keywords:** maize diversity, artificial selection, Maize SNP50K BeadChip, maize genomics

## Abstract

In Mexico's territory, the center of origin and domestication of maize (*Zea mays*), there is a large phenotypic diversity of this crop. This diversity has been classified into “landraces.” Previous studies have reported that genomic variation in Mexican maize is better explained by environmental factors, particularly those related with altitude, than by landrace. Still, landraces are extensively used by agronomists, who recognize them as stable and discriminatory categories for the classification of samples. In order to investigate the genomic foundation of maize landraces, we analyzed genomic data (35,909 SNPs from Illumina MaizeSNP50 BeadChip) obtained from 50 samples representing five maize landraces (Comiteco, Conejo, Tehua, Zapalote Grande, and Zapalote Chico), and searched for markers suitable for landrace assignment. Landrace clusters could not be identified taking all the genomic information, but they become manifest taking only a subset of SNPs with high *F*_*ST*_ among landraces. Discriminant analysis of principal components was conducted to classify samples using SNP data. Two classification analyses were done, first classifying samples by landrace and then by altitude category. Through this classification method, we identified 20 landrace-informative SNPs and 14 altitude-informative SNPs, with only 6 SNPs in common for both analyses. These results show that Mexican maize phenotypic diversity can be classified in landraces using a small number of genomic markers, given the fact that landrace genomic diversity is influenced by environmental factors as well as artificial selection due to bio-cultural practices.

## 1. Introduction

Maize (*Zea mays*) is grown in most of Mexico under contrasting environmental conditions, from tropical rainforests to arid semideserts, and by farmers from a variety of cultural backgrounds (Boege, 2008; Ruiz Corral et al., 2013). Therefore, the phenotypic and genetic diversity of maize landraces is influenced by a multiplicity of environmental and bio-cultural factors. Environmental factors are mainly correlated to temperature and rainfall, while bio-cultural variables relate to socioeconomic factors and the distribution of ethnic diversity (Boege, 2008; Perales and Golicher, 2014). As a consequence, there is a large diversity within and among landraces. This generates doubts about the genomic basis for landrace classification, even though maize racial categories are widely used by agronomists. Here, we aim to distinguish between natural and artificial selection as drivers of genomic variation among landraces. This is a challenge rarely addressed, but necessary for safeguarding maize diversity considering the factors affecting maize genetic diversity, as well as for rationally guiding crop improvement in the face of changing environmental conditions.

Maize landraces can be defined as dynamic populations with a historical origin and distinct identity, and which are often genetically diverse, locally adapted, and associated with a set of farmers' practices of seed selection and field management as well as with traditional knowledge (Camacho-Villa et al., 2015). Mexican maize diversity was described and classified into landraces during the twentieth century. Nowadays, CONABIO (Mexican National Commission for the Knowledge and use of Biodiversity) recognizes 64 landraces existing in Mexico, clustered in seven racial groups or complexes (CONABIO, 2011).

Recent studies on Mexican maize genomic diversity have found that the distribution of genetic variation is better explained by environmental variables governed by altitude and latitude than by landrace identity (van Heerwaarden et al., 2011; Breña Ochoa, 2013; Arteaga et al., 2015; Romero Navarro et al., 2017). Maize is grown throughout Mexico along an altitude gradient spanning 0–2,700 masl. This wide range in altitude correlates with a substantial range in temperature and moisture gradients, which are associated with local adaptation of maize populations growing at different altitudes (Pressoir and Berthaud, 2004; Mercer et al., 2008; Ruiz-Corral et al., 2008; Romero Navarro et al., 2017). Agronomically, local adaptation implies high levels of genotype-by-environment interactions, making it difficult to grow landraces in altitudes different from their original ones (Perales et al., 2003; Mercer et al., 2008; Lorant et al., 2017). Another factor influencing altitudinal differentiation is gene flow between domestic maize and populations of its wild ancestors (teosintes *Z. m*. ssp. *mexicana* and *Z. m*. ssp. *parviglumis*). Teosinte subspecies have allopatric distributions, *Z. m*. ssp. *mexicana* growing in highlands and *Z. m*. ssp. *parviglumis* growing in lowlands. Highland maize populations' genomic constitution is notably influenced by teosinte *mexicana* growing sympatrically, as there are high levels of gene flow among them (van Heerwaarden et al., 2011; Hufford et al., 2013; Lorant et al., 2017; Romero Navarro et al., 2017). Taken together, these facts account for the correlation between genetic variation and altitude.

Morphologically different populations of maize present low levels of genotypic differentiation, revealing that phenotypic and genetic variation do not correlate (Pressoir and Berthaud, 2004; Arteaga et al., 2015). This fact suggests that phenotypic diversity in maize is the result of a complex process and cannot be explained only by genetic factors. Genetic homogeneity among maize populations is explained by the fact that these populations are open systems with high levels of gene flow through open pollination and seed exchange by farmers (Dyer and Taylor, 2008; Orozco-Ramírez et al., 2016). Still, the high degree of variability within and among landraces is associated with the selection by smallholders of different parts of the plant, the different environmental conditions in which it is grown, and in general the different agroecological practices people growing maize have (Bellon et al., 2009; Orozco-Ramírez et al., 2016). Despite the complex genetic constitution of maize populations, landraces are phenotypically distinguishable, suggesting that there must be a genetic basis underlying phenotypic diversity. This apparent paradox could be explained by farmers selecting for genes with major and pleiotropic effects (Pressoir and Berthaud, 2004; Bouchet et al., 2017). Therefore, identifying the genetic variations underlying landrace phenotypic differences has proved a complicated task.

Currently, smallholder cultivators growing maize select seeds year by year to meet quality and variety desires according to their diet and traditions (Ortega Paczka, 2003; Boege, 2008; Orozco-Ramírez et al., 2016). This has been done for over 9,000 years by Mexican indigenous groups and mestizo subsistence producers (Yamasaki et al., 2005; van Heerwaarden et al., 2011). Nowadays, Mexican maize landraces are still grown mostly by smallholders, typically in parcels of land <5 ha in marginal environments, but together accounting for 85% of Mexico's productive land (SIAP, 2008; Arteaga et al., 2015). Because of this joint extent of farmland, and because maize landraces often equal or surpass the yield production of breeding lines in marginal environments (Perales et al., 2003), maize landraces are crucial for Mexican food security (Bellon et al., 2011; CONABIO, 2011). Maize landraces have also historically been used as donor material for breeding efforts at a global scale, making them important reservoirs of genetic diversity for further improvement (Troyer, 2004; Smith et al., 2015; Dwivedi et al., 2016).

We believe that careful examination of genomic variation between landraces is a way to test for evidence of the influence of bio-cultural processes (human selection of desired characters based on cultural preferences) that may not have been detected by less comprehensive approaches of earlier studies. This could also grant the distinction of loci influenced by human selection from those influenced by natural selection (given by environmental conditions). In the present study, we used SNP genomic information from 50 samples of five Mexican maize landraces to create a classification model based on landrace identity. This allowed the identification of informative SNPs for sample landrace classification. SNPs influenced by environmental factors derived from altitude differences were also identified. In this way, the influence of environmental influence could be discriminated from landrace-informative SNPs, thus allowing to pinpoint probable artificially selected loci distinguishing landraces.

## 2. Materials and methods

### 2.1. Sampling and SNP genotyping

**Figure 1:**
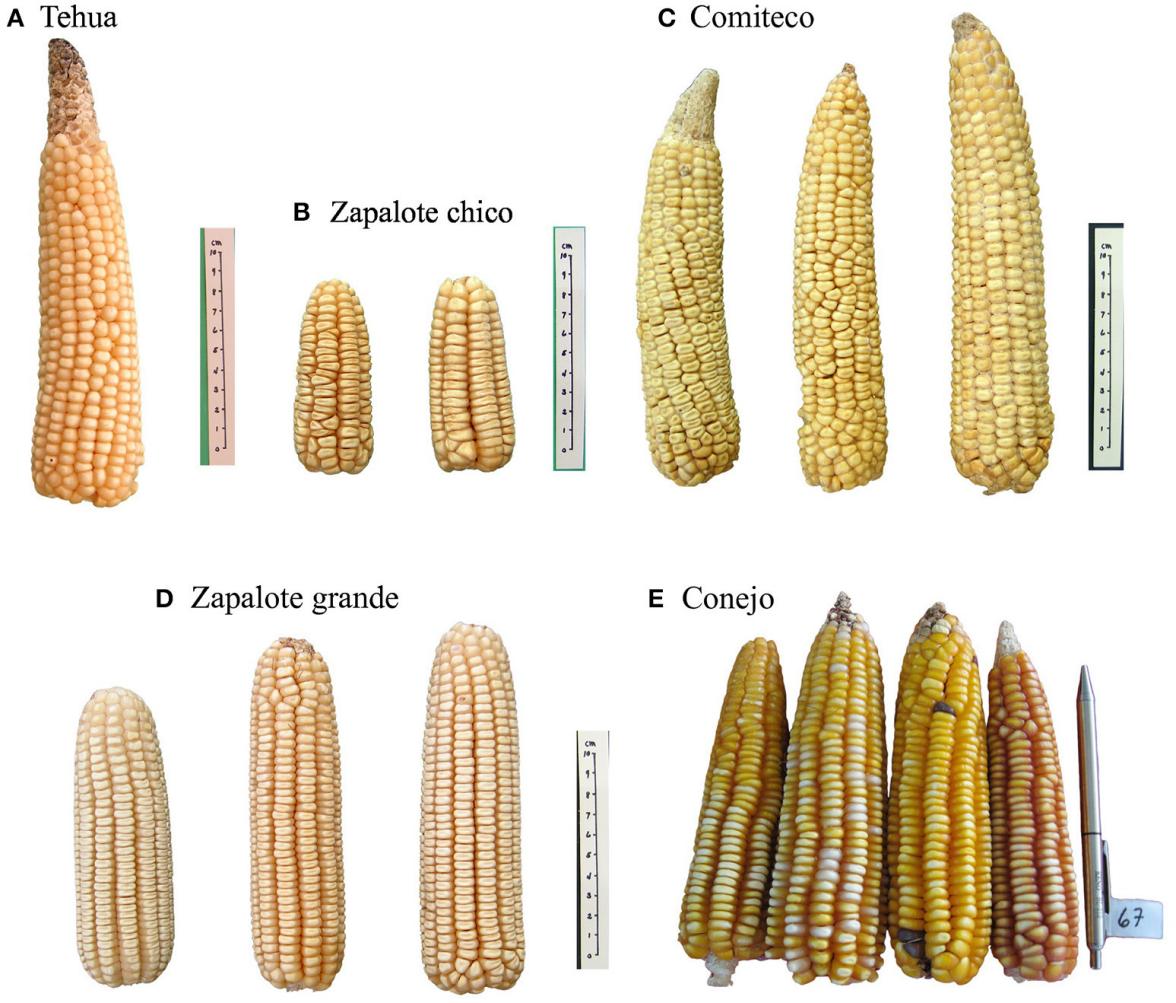
**Representative images of each of the landraces used in this study, note the difference in cob size among them**. Images credit: Instituto Nacional de Investigaciones Forestales Agrcolas y Pecuarias (INIFAP) and Global Maize Project (CONABIO, 2011).

**Figure 2:**
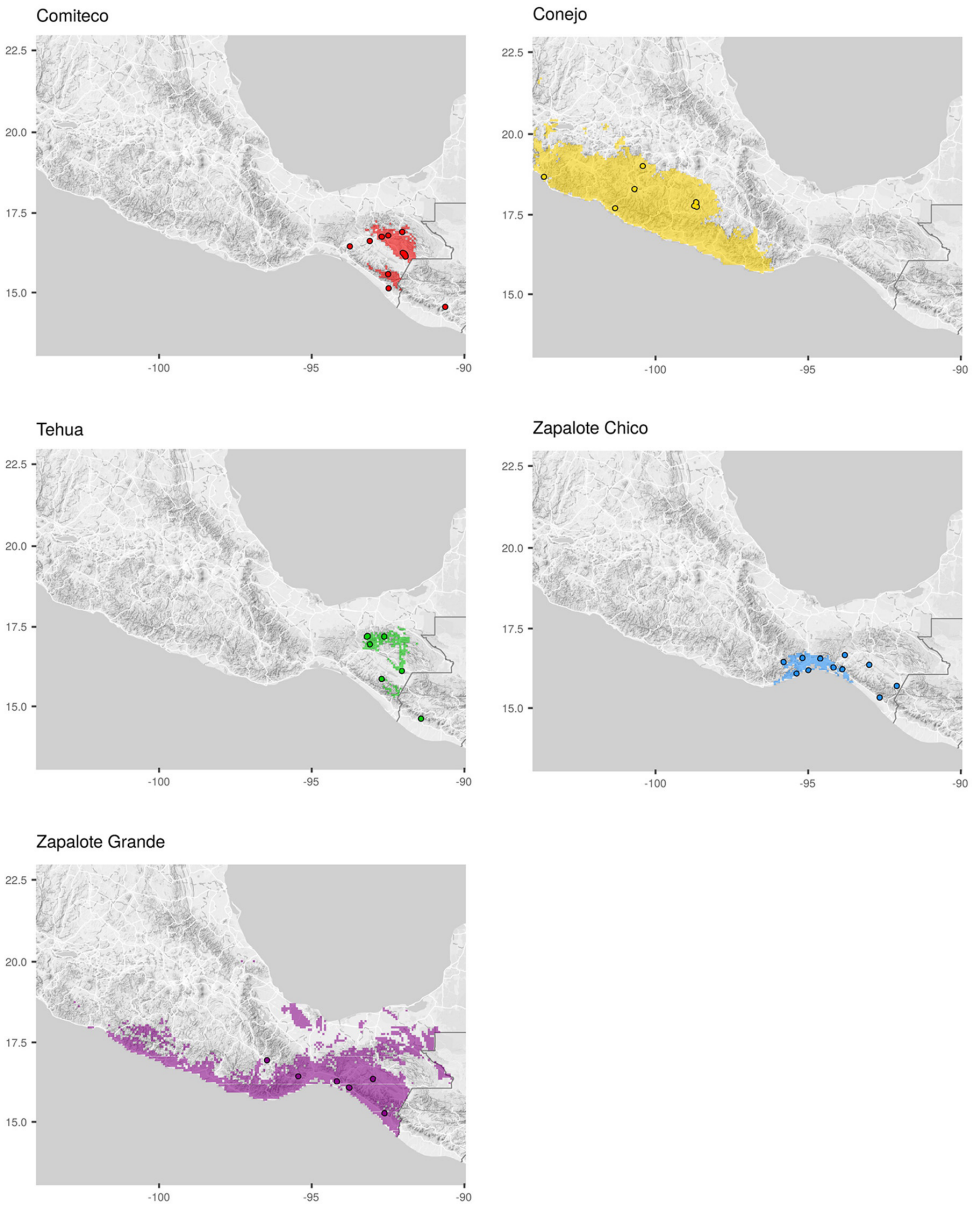
**Sample collection sites**. Each map shows the potential distribution of the maize landrace (shadowed area) and the location where the genotyped accessions of each landrace were collected (dots). Landrace potential distributions were taken from Perales and Golicher (2011).

The 59 Mexican landraces defined by CONABIO (2011) (http://www.biodiversidad.gob.mx/usos/maices/razas2012.html) were examined to select five (Zapalote Chico, Conejo, Zapalote Grande, Tehua, and Comiteco) that show contrasting phenotypic characteristics (Figure 1 and Table 1). These landraces are represented by 37 accessions from the Germplasm Bank of the International Maize and Wheat Improvement Center (CIMMYT) that were genotyped in this study (see below), plus 13 accessions from a previous study that used the same extraction protocol and SNP data generation (Arteaga et al., 2015). Our sample size for each landrace was 8–14 accessions, collected between 1946 and 2010 and covering most of the potential distribution of each landrace (see Supplementary Table 1 and Figure 2). Potential distributions were taken from Perales and Golicher (2011).

**Table 1:** **Main phenotypic characteristics of the analyzed landraces according to CONABIO (2011)**.

**Landrace**	**Zapalote Chico**	**Conejo**	**Zapalote Grande**	**Comiteco**	**Tehua**
Racial group	Tropicales precoces (Tropical precocious)	Tropicales precoces (Tropical precocious)	Dentados tropicales (Tropical dents)	Maduración tardía (Late maturation)	Maduración tardía (Late maturation)
Plant	Very short (1–2 m), few leaves	Short (1.2–1.9 m)	Short to intermediate, medium number of leaves	Very tall (4–5 m), many leaves	Very tall (up to 6 m), many leaves
Maturation time	Early	Early	Intermediate	Very late	Very late
Cob	Small	Small, 8–10 rows	Small	Large	Large
Grain type	Small, dent	Medium, flint/semident	Small, dent	Large, dent/semiflint	Large, dent/semiflint
Samples altitude range (masl)	5–700	180–1,685	8–1,000	100–2,241	122–1,604

From CIMMYT's accessions, one seed per accession was germinated in greenhouse conditions. DNA was extracted from leaf tips collected from 3-week-old seedlings with Thermo Fisher Scientific Genomic DNA Purification Kit (K0512) (Thermo-Fisher Scientific, Waltham, MA, USA). DNA quality was determined by agarose gel electrophoresis and with a Thermo-Fisher Scientific Nanodrop Spectrophotometer 2000 (Thermo-Fisher Scientific, Waltham, MA, USA). Accessions with a 260/280 ratio below 1.6 were re-extracted.

Single nucleotide polymorphisms were genotyped at INMEGEN (Instituto Nacional de Medicina Genómica) with the Illumina MaizeSNP50 BeadChip on an Infinium HD assay (Illumina, San Diego, Ca, USA). Automated allele calling was done in GenomeStudio 2010.1 (Genotyping module 1.7.4; Illumina); loci with GenTrain score <0.3 and with more than 30% missing data were excluded. Data was exported to PLINK format (Purcell et al., 2007). A maximum of 10% missing data per individual was allowed. Data from the 13 accessions obtained from Arteaga et al. (2015) was added to this dataset. Only SNPs present in both groups of accessions were retained.

### 2.2. Subsetting SNPs into candidate loci of artificial selection

In order to guide the search for landrace-distinguishing SNPs, three sets of SNPs were defined: (1) all the SNPs available (35,909), (2) SNPs of domestication and improvement (974 SNPs), and (3) SNPs with high *F*_*ST*_ (435 SNPs). Set one refers to all the SNPs retained after filtering and joining our dataset with Arteaga's. The SNPs of domestication and improvement were determined using the maize domestication and improvement genes reported by Hufford et al. (2012) and Meyer and Purugganan (2013). To do this we used the Panzea Genotype Search Tool (http://cbsuss05.tc.cornell.edu/hdf5/hdf5.asp) to search which of our 35,909 SNPs were located in loci reported by Hufford et al. (2012) and Meyer and Purugganan (2013). SNPs with high *F*_*ST*_ were defined after calculating the value of *F*_*ST*_ among landraces for every SNP using the SNPStats package (Clayton, 2015) in R (R Core Team, 2008) and taking the 1% of SNPs with the highest *F*_*ST*_.

### 2.3. Identifying loci to distinguish landraces

We used the three previously defined set of SNPs to examine if they could be useful for distinguishing the landraces in a principal components analysis (PCA) and discriminant analysis of principal components (DPCA). The PCA was performed for each set using the package SNPRelate (Zheng et al., 2012). The first two components were used for plotting, coloring accessions by landrace. A K-means clustering analysis was performed to the three sets of SNPs to identify which set better recovered the landrace pattern using the packages adegenet (Jombart, 2008) and ape (Paradis et al., 2004). The DAPC (Jombart et al., 2010) was carried out to classify samples according to their landrace. This analysis was performed with the set of SNPs with high *F*_*ST*_ using the R package adegenet. The contribution of each SNP to the DAPC classification model was calculated. The SNP distribution showed a long-tailed distribution, the top contributing SNPs (~2%) were identified as landrace-informative SNPs.

### 2.4. Identifying loci associated to altitude

To discard the possible confounding effect of altitude in the SNPs identified as landrace-informative, we repeated the previous analyses but grouping the accessions by altitude instead of landrace. For this, first the accessions were grouped according to their altitude in two groups: high altitude (>750 masl) and low altitude (0-750 masl). The limit between the altitudinal groups was defined following the altitudinal distribution of the landraces included, separating the interquartile range of the landraces typically growing at low altitude (Zapalote Chico and Zapalote Grande) from the interquantile range of the landraces growing at high altitude (Tehua and Comiteco). Still, all landraces except Zapalote Chico had accessions from both altitude categories. All Zapalote Chico accessions belonged to the low altitude group (see Table 1).

*F*_*ST*_ index of each SNP between these two altitude groups was calculated using the SNPStats R package, and a subset of the 1% of SNPs with highest *F*_*ST*_ was extracted to continue the analysis. Using this group of SNPs, a DAPC was performed as earlier, but so as to have a model that classifies the samples according to their altitude group. The contribution of each SNP to the classification model was obtained, and the SNPs with the highest contribution were identified as altitude-informative SNPs. The groups of landrace informative SNPs and altitude associated SNPs were compared to identify which landrace-informative SNPs are independent from altitude.

### 2.5. Biological annotation of obtained SNPs

SNPs identified as landrace- or altitude-informative were reviewed in order to obtain their genomic position and biological annotation. This was accomplished by searching for the corresponding loci through the locus lookup tool at MaizeGDB (www.maizegdb.org/locus_lookup; Andorf et al., 2010). Further information about genes associated with the obtained SNPs was recovered through literature review.

### 2.6. Data and code accessibility

All SNP data and R scripts used for this study is available in the Dryad online repository: http://dx.doi.org/10.5061/dryad.j2n8q.

## 3. Results

### 3.1. Subsetting SNPs into candidate loci of artificial selection

After data filtering, the final SNPs data set consisted of 35,909 SNPs. The domestication and improvement SNP set consists of 974 SNPs located within the loci associated with domestication and improvement reported by Hufford et al. (2012) and Meyer and Purugganan (2013). A total of 84 SNPs were located in the loci reported by Meyer and Purugganan. In the loci reported by Hufford et al. 501 SNPs were located in loci associated with improvement and 389 SNPs in loci associated with domestication.

**Figure 3:**
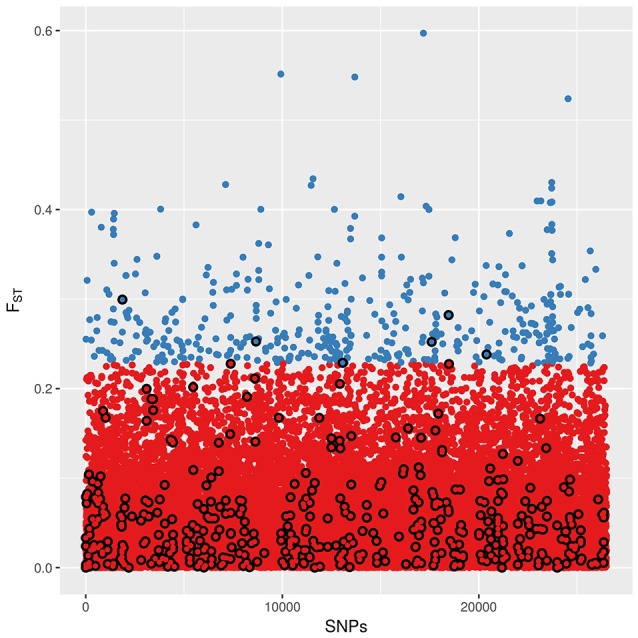
**Distribution of SNPs according to their ***F***_***ST***_ value among landraces**. Blue: SNPs set of high *F*_*ST*_ SNPs. Black circle line: SNPs of the domestication and improvement. There are only six SNPs belonging to both SNP sets.

Values of *F*_*ST*_ among landraces for every SNP ranged from 0 to 0.597 (mean = 0.056, *SD* = 0.055). Most SNPs present *F*_*ST*_ values close to 0, indicating that they are poorly differentiated among landraces. The high *F*_*ST*_ SNP set consists of 435 SNPs with *F*_*ST*_ values higher than 0.2283, representing 1% of SNPs with highest *F*_*ST*_ (Figure 3). Most domestication and improvement SNPs have relatively small values of *F*_*ST*_, and only 6 SNPs are common between the two sets.

### 3.2. Identifying loci to distinguish landraces

**Figure 4:**
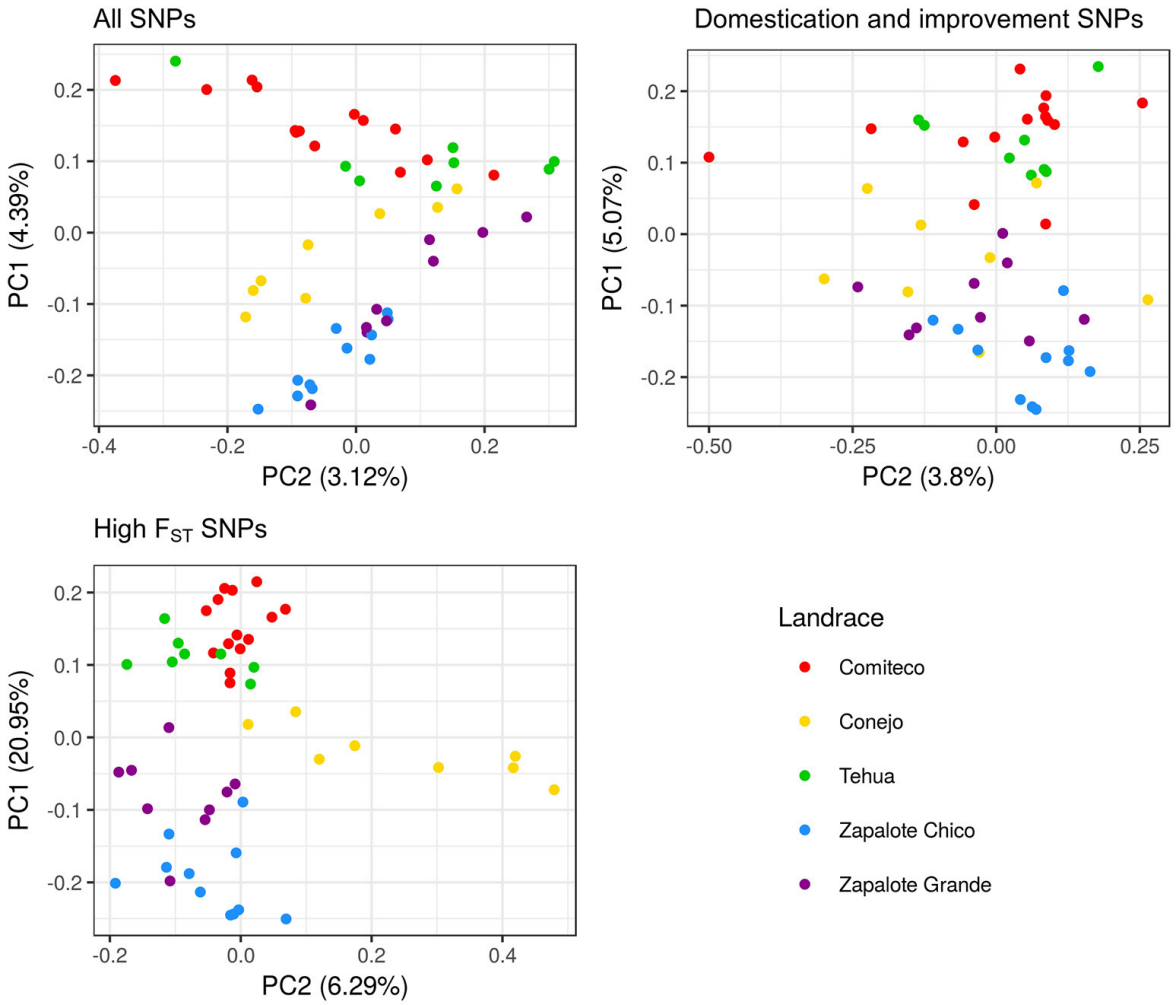
**PCA plot for the first two principal components using different SNP sets**.

PCA using each of the three sets of SNPs reflects that the genomic information each set carries is structured differently. Figure 4 shows the different distribution patterns of the first two principal component among the three SNP sets. The percent of variance explained by the first three PCs of the high *F*_*ST*_ SNP set is 20.91, 6.30, and 5.67, respectively, which is higher than the variance explained by the first three PCs of the set of all SNPs (4.39, 3.12, 2.89) and the domestication and improvement SNPs (5.04, 3.80, 3.60).

**Figure 5:**
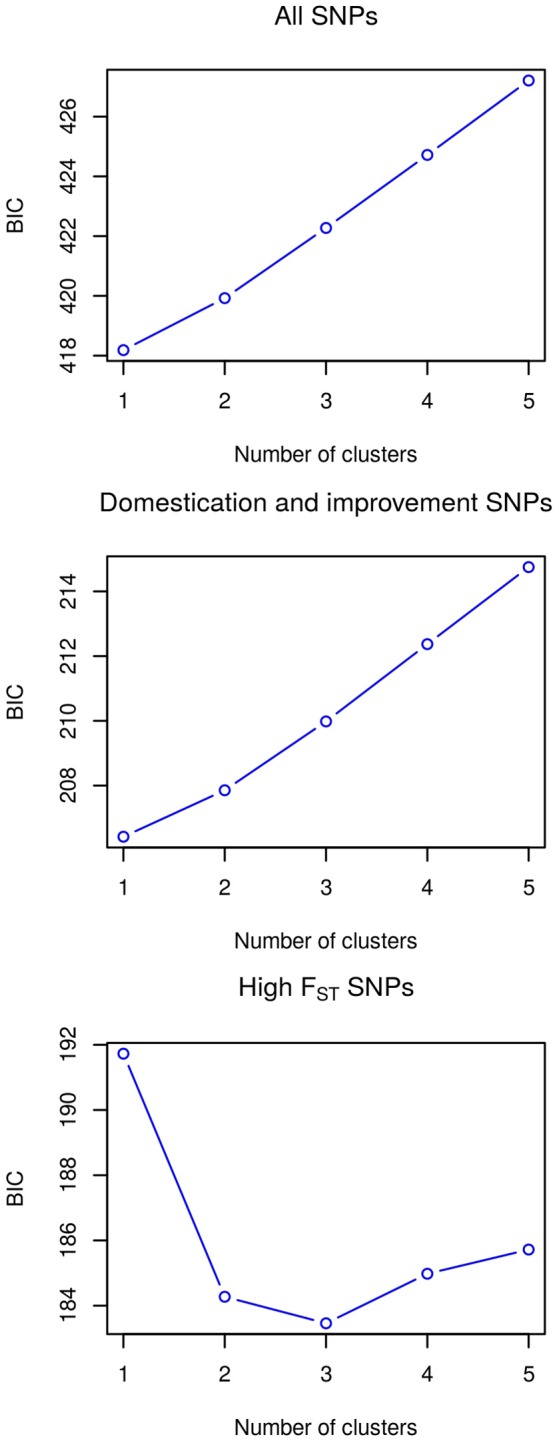
**Clustering analysis with the three SNP sets, the lower value of BIC indicates optimal clustering**.

The utility of the three sets of SNPs for grouping the accessions according to their landrace was assessed by a *k*-means clustering analysis, testing from one to five clusters and determining the appropriate number of clusters through the Bayesian information criterion (BIC) (Figure 5). For the all SNPS and domestication and improvement SNPs sets, the best clustering groups all the accessions into a single cluster. The best clustering for the high *F*_*ST*_ SNP set identifies three groups: one group containing only accessions of Zapalote Chico, a second group with accessions of landraces Zapalote Grande and Conejo, and a third group with accessions of landraces Tehua and Comiteco. Since the only SNP set capable of identifying different clusters in the accessions was the high *F*_*ST*_ SNP set, we continued the analysis only with these SNPs.

**Figure 6:**
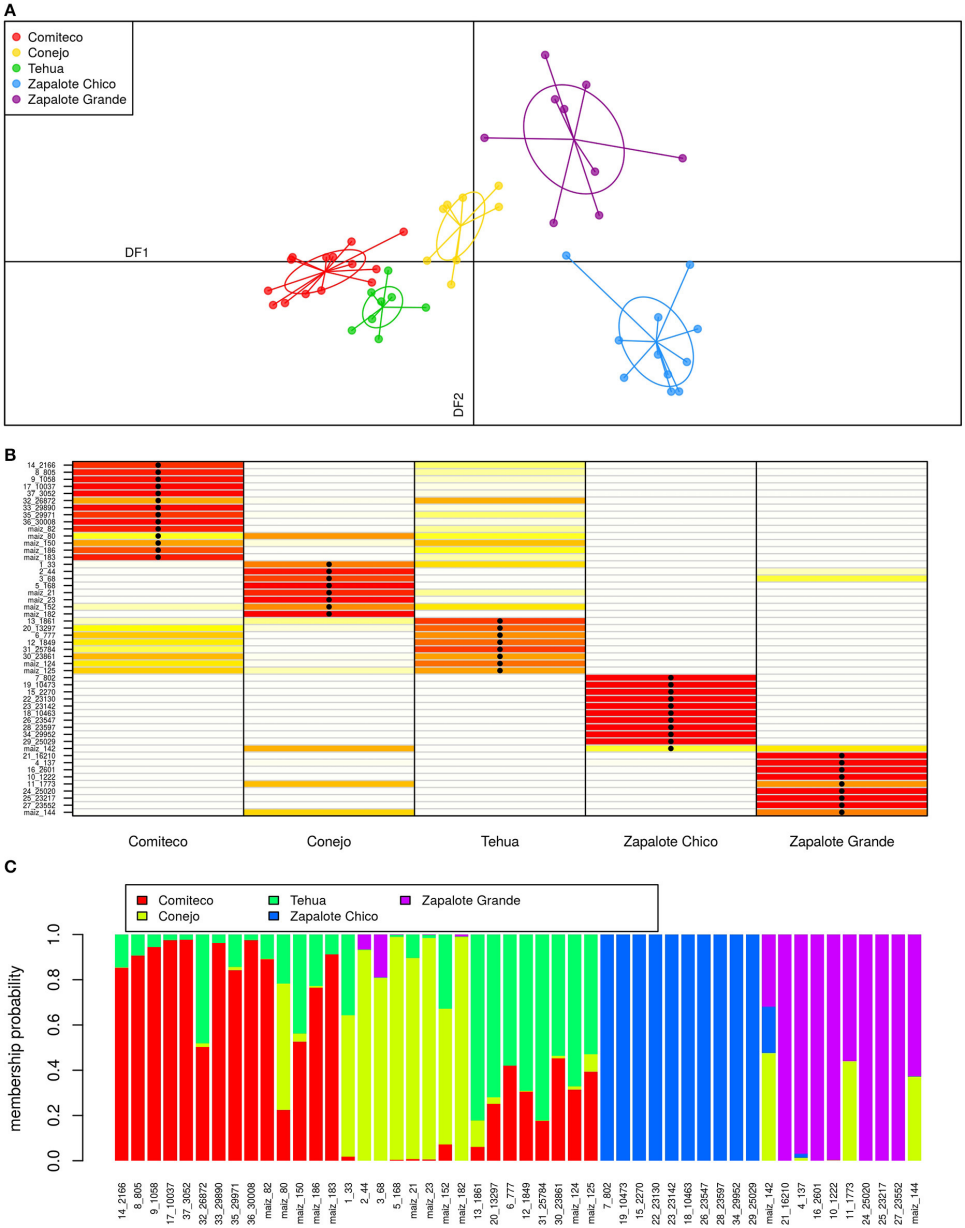
**Landrace DAPC. (A)** Classification plot: here the two discriminant functions of the DAPC are plotted for each sample. **(B)** Assignment plot: this plot shows the accuracy of the model to recover samples landrace. The color of each cell points out the probability of assigning a given sample (row) to the corresponding landrace (column) with red colors meaning high probability of assignment. The black dots in cells show the assigned landrace of the sample, so when a dot falls on a red cell means a correct landrace classification. **(C)** Structure plot: this admixture-like plot describes, for each sample, the membership probability to the different landraces according to the model.

The classification model from the DAPC analysis used the first four principal components of the high *F*_*ST*_ SNP set and was able to classify the accessions using two discriminant functions (Figure 6A). The first discriminant function (horizontal axis) recovers an altitude distribution pattern for the accessions, sorting them successively from the landrace growing in the lowest altitudes (Zapalote Chico) to the landrace growing in the highest places (Comiteco). The second discriminant function separates races growing in similar altitudes. The two discriminant functions are able to separate the clusters of accessions according to their landrace.

This model has a high degree of accuracy in assigning the correct landrace to each sample, 94% of the samples are correctly classified (Figure 6B). This accuracy is achieved despite the high degree of admixture present among some samples (Figure 6C). Tehua and Comiteco are the two landraces with more admixture between them; and accordingly, the ones in which the landrace reassignment by the model is less discriminant. Samples from Conejo and Zapalote Grande show certain degree of admixture, but the model is able to reassign their accessions accurately. Zapalote Chico is the landrace with less admixture, forming a clearly separated cluster from the rest. This classification model gains more validity considering that the samples we analyzed are representative of each landrace's geographic distribution and their time of collection spans from 1946 to 2010.

The contribution of each SNP to each discriminant function was calculated. For both functions, the SNP contributions showed an exponential distribution, with most SNPs having very little contribution and a few SNPs with very high contribution (Supplementary Figure 1). The top contributing SNPs of each function were identified, giving a group of 11 informative SNPs for the first function and 9 informative SNPs for the second function (Supplementary Table 2). Together, these 20 SNPs are identified as the landrace-informative SNPs. Genomic position and biological annotation of these SNPs was obtained: 9/20 are associated to seven genes, four of which have not yet been characterized. The three characterized genes are a polygalacturonase enzyme, heat shock factor 30 (HSF30), and transcription factor IIIA (Supplementary Table 2).

### 3.3. Identifying loci associated with altitude

A set of SNPs differentiated between the two altitude groups was identified using *F*_*ST*_ index values. *F*_*ST*_ values between altitude groups for all SNPs ranged from 0 to 0.5251 (mean = 0.0345, *SD* = 0.043). Data for the 1% of SNPs with the highest *F*_*ST*_ (above 0.1728) was extracted. The best k-means grouping using this group of SNPs resulted in two groups, which almost perfectly recovered the high and low altitude groups previously defined, clustering only one accession incorrectly. The DAPC was done using the first principal component and one discriminant function (Supplementary Figure 2). The classification model had an almost perfect performance, misclassifying only that same accession that was incorrectly clustered before (Figure 6C). The contribution of each SNP to the discriminant function followed an exponential distribution. The top 14 contributing SNPs were identified as altitude-informative SNPs (Supplementary Figure 3), and their genomic position and biological annotation was obtained (Supplementary Table 3). Of the 14 altitude associated SNPs, eight fall within loci of genes of which only three genes have been annotated. These genes correspond to the auxin responsive protein SAUR40, Transformer-2, and the heat shock factor 30 (HSF30).

Comparing the altitude-informative SNPs with the landrace-informative SNPs, there are five SNPs shared between the two sets (Supplementary Table 2), two of which are associated to an uncharacterized gene (GRMZM2G100103). Additionally, both analyses identified a SNP, albeit a different one each, part of a gene associated with the heat shock factor protein 30. So this locus was also considered to be shared between the analyses. In total, for the landrace analysis there are 14 uniquely informative SNPs and six SNPs shared with the altitude analysis. For the altitude analysis, there are eight unique SNPs that are not shared with the landrace analysis.

## 4. Discussion

We found that samples of Mexican maize can be classified by landrace using only a small number of SNPs. Furthermore, we could identify genomic loci differentiating the five landraces we studied, and we could distinguish them from loci correlated with altitude.

### 4.1. Clustering by landrace is achieved only with high *F*_*ST*_ SNPs

Both the PCA and the k-means clustering analysis show that accession clustering depends on the set of SNPs used. In particular, the set of SNPs with high *F*_*ST*_ groups the samples according to their landrace while neither of the other two sets (“domestication and improvement SNPs” and “ all SNPs”) does.

The k-means clustering analysis grouped all the samples in a single cluster when using all the SNPs, meaning that the whole-genomic distribution of variation is not structured by landrace. In other words, from a whole-genome perspective, these five landraces are not distinguishable among them. This is consistent with previous studies showing that maize genomic structure is not explained by landrace, and is better correlated with environmental factors (van Heerwaarden et al., 2011; Breña Ochoa, 2013; Arteaga et al., 2015). Clustering analysis with the domestication and improvement SNPs also failed in finding structure among the samples, grouping them all in one cluster. Domestication SNPs are defined as loci with differentiated between teosinte and maize, thus involved in maize domestication from its wild relative. In this sense, it is expected that domestication SNPs are not differentiated among maize landraces. On the other hand, the improvement SNPs we used were found as loci differentiated between maize landraces and modern improved lines (Hufford et al., 2012). Modern improved lines derive from distinct landrace founders and have been subject to subsequent selection for agronomic traits, which are not the same that constitute the differences among these landraces. Thus, genomic variation in modern lines not necessarily maintains the structure of variation found on landraces, as shown by the low values of *F*_*ST*_ found in most domestication and improvement SNPs (Figure 3) indicates that these loci are not differentiated among landraces and so do not explain morphological differentiation.

Using the set of high *F*_*ST*_ SNPs (435 SNPs, Figure 2) recovers the landrace assemblages as recognized by morphology. This suggests that this set of SNPs are under different selective pressures depending on the landrace, therefore varying in a different way from the rest of the genome. These selective pressures are probably a combination of environmental differences among the areas the landraces are grown, and those guided by the smallholder farmers that continuously select seed looking for certain characteristics of the crop: desired morphology, taste, compatibility with their agricultural practices, among others (Pressoir and Berthaud, 2004; Boege, 2008; Bellon et al., 2011).

We were able to classify samples by landrace using the high *F*_*ST*_ SNPs. Sample classification was achieved with high accuracy (94%) despite broad landrace admixture. Most samples show some degree of mixing among landraces (Figure 6C), this pattern is explained by the known high degree of gene flow among maize population. There is a high degree of gene flow in maize populations. It is caused both by open pollination of maize fields flowering at the same time, and by seed transportation and exchange by farmers (Dyer and Taylor, 2008; Orozco-Ramírez et al., 2016). Gene flow among landraces is also strengthened by the fact that 85.5% of the total average surface cultivated with maize in Mexico (covering an average of 8.4 million ha between 1996 and 2006) is cultivated by smallholders using landraces (SIAP, 2008, 2016) therefore creating a large and genetically diverse landscape for open pollination to occur. Seed manipulation performed by producers maintains a phenotypic differentiation of maize landraces, in spite of high levels of gene flow, both by pollen and seed exchange (Pressoir and Berthaud, 2004; Dyer and Taylor, 2008; Mercer et al., 2008; Orozco-Ramírez et al., 2016).

### 4.2. Distinguishing the influence of artificial from natural selection on the genome

Accurate models to classify accessions by landraces and altitude categories were attained through DAPC, allowing for the further inference of informative markers for both models. Informative SNPs were identified by their high contribution to the classification models, being the most informative SNPs when trying to classify by race or by altitude. SNPs association to landrace and altitude is based on the idea that if the model found these marker to be the most useful when separating the landrace/altitude groups, it is because there are underlying differences among groups in these markers, probably due to selection. We identified 14 landrace-informative SNPs that are independent from altitude. Even though our altitude analysis does not account for all the environmental factors, previous studies establish altitude as the principal factor shaping maize genomic variation (van Heerwaarden et al., 2011; Breña Ochoa, 2013; Arteaga et al., 2015; Romero Navarro et al., 2017), thus making it a valid approximation of environmentally driven natural selection.

A potential caveat of our analyses is that the genomic data used was obtained from the Illumina MaizeSNP50 BeadChip. This SNP chip was developed from the genome of maize B73 line to identify genomic sites of agronomic interest (Ganal et al., 2011). Therefore, it presents a bias toward identifying variation present in the B73 genome, and it is highly probable that there are other genomic regions differentiated among landraces that are not recognized by the SNP chip. In other words, our analysis may be missing variation at other alleles, which make up a substantial proportion of natural variation (Huang and Han, 2014).

The proposed landrace and altitude informative loci identified here represent an opportunity to further confirm if SNPs identified by genomic analyses can explain landraces phenotypic characteristics. This can be assessed by testing their direct influence in the plant phenotype, and analyzing their variation among other maize landraces. However, through searching for the functional annotation of associated genes, our approximation showed interesting results hinting functional importance of the identified loci.

One of the five annotated genes associated with the informative markers, the heat shock factor 30 HSF30, was shared between the landrace and the altitude analyses. HSF30 is part of the heat shock transcription factors that drive the cellular heat stress response in plants (Westerheide et al., 2012). HSF30 has not been functionally characterized in maize, but it is homologous to heat shock factor A2 present in other plant species where its function has been studied. For example, in *Arabidopsis thaliana*, HSF2A is the heat shock factor with highest expression under heat stress conditions, and acts as regulator of several stress-responsive genes (Schramm et al., 2006); and in *Solanum lycopersicum*, it has been shown that HSF2A transcription is strictly dependent on heat stress, and acts as a strong transcriptional activator for heat shock proteins (Nover et al., 2001). Considering the homology between *HSF30* and *HSF2A* and the relationship between altitude and temperature, our results suggest that landraces hold a component of local adaptation to the altitudinal range where they are grown.

The other two annotated genes identified among the landrace-informative SNPs correspond to a polygalacturonase enzyme and transcription factor IIIA. An homologous gene of transcription factor IIIA has been characterized in rice, where its function was associated with plant development and tolerance to abiotic stress related to salinity, low temperatures, and drought (Huang et al., 2012). We consider transcription factor IIIA of special interest, because of the non-linear phenotypic effects a transcription factor can exert on a developing plant in the context of a transcription regulatory network (Davila-Velderrain and Alvarez-Buylla, 2014). Further maize research focusing on the effects of variation in this gene grown under different environmental conditions would be very interesting.

### 4.3. What makes zapalote chico so different and homogeneous?

An interesting result in the classification model is that the samples of Zapalote Chico were the most distant cluster and presented a high homogeneity within the landrace (Figure 6). This has already been highlighted by a study focusing in the role of MuDR transposons, which are a characteristic of the Mutator maize line and are associated with a high rate of somatic mutations (Gutiérrez-Nava et al., 1998). Specifically, Gutierrez-Nava and collaborators reported that Zapalote Chico has an elevated rate of MuDR transposons, and that, when inter-crossed with germplasm from another landrace, the genomic elements present in Zapalote Chico cause somatic mutations, an effect known as hybrid dysgenesis. These mutations do not happen when two samples of Zapalote Chico are inter-crossed, showing that somehow the mutagenic activity of the transposons is repressed. This phenomenon explains Zapalote Chico's genetic isolation relative to other landraces. Interestingly, Zapotecs, the indigenous people growing this landrace, interpret this phenomenon in the form of a myth, which holds that when Zapalote Chico is crossed with another kind of maize it “kills" it (Gutiérrez-Nava et al., 1998).

### 4.4. Genetic differences are not enough to explain the landraces existence

In this study, we focused on genomic differences among maize landraces; we did not consider other factors that cause phenotypic differences and that could have a strong influence on crop phenotypes. Notably, the manipulation of the agroecological system by the people that grow maize is an important source of variation that could explain part of the differences seen among landraces (Jardón Barbolla and Benítez, 2012). Nonetheless, landraces, characterized in terms of both local adaptation and satisfaction of cultural preferences, have an associated genomic variation underlying their differences (Bellon et al., 2009). Several tens of millions of rural populations in Mexico depend solely on their maize production. Mexican cultural richness is reflected in the wide differences of uses people give to maize, resulting in a great variety in terms of flavor, shape, color, texture, and other organoleptic characteristics (Boege, 2008). Although natural and artificial selection exert their influence in particular loci, the concerted action of whole genomic variation and external conditions during a plant's development determine its phenotype, and consequently, overall success for its further sowing by the people using it.

Mexican maize is grown in complex agroecological systems where biological (interspecies relations, genetic flow, pollinators activities, soil microbiome, etc.), social, and cultural (farming techniques, seed interchange, land tenure patterns, religious, and symbolic uses, etc.) factors interact. This demands the study of maize and the agroecosystems in which it grows, including the groups of small-scale maize producers, as a whole for the understanding of maize diversity.

Mexican maize landraces contain a high genomic diversity within and among them, which results from the underlying multiplicity of environmental and biocultural factors mediating their existence (Arteaga et al., 2015). These factors entail selective pressures, both natural/environmental and artificial/biocultural, acting on different genomic regions. In this study we found that, even though from a whole-genome perspective maize landraces are indistinguishable, it is possible to identify genomic regions differentiated among landraces. Furthermore, combining the landrace analysis with an environmental association analysis on the genomic data, it is possible to recognize loci influenced by natural or artificial selection. Our results are evidence of the genomic variation that exists in maize landraces and their importance for maize diversity conservation. Nevertheless, it is important to keep in mind that landraces are evolving entities, sustained through a process based on a great genomic, environmental and bio-cultural diversity. Furthermore, focusing on preserving landraces by monitoring their phenotypic diversity through a small number of representative samples may lead to losing the genetic diversity underlying local adaptation. Understanding the genomic foundation of landrace diversity and the processes influencing it is imperative for its maintenance. Given the different factors shaping maize biodiversity, a program aimed at conserving the genomic and phenotypic richness of maize requires the maintenance of the biocultural processes in which it is grown and used.

## Author contributions

AW, JC, and DP designed the study. AW coordinated the study. JC conducted lab work. JC and AM designed and conducted the analyses. All authors analyzed the results and wrote the manuscript.

## Funding

Funding was provided by the Dirección General del Sector Primario y Recursos Naturales Renovables (DGSPRNR) of the Secretaría de Medio Ambiente y Recursos Naturales (SEMARNAT) through the grants 2009-1 and 2013 to the Comisión Nacional para el Conocimiento y Uso de la Biodiversidad (CONABIO).

### Conflict of interest statement

The authors declare that the research was conducted in the absence of any commercial or financial relationships that could be construed as a potential conflict of interest. The reviewer SR and handling Editor declared their shared affiliation, and the handling Editor states that the process nevertheless met the standards of a fair and objective review.
