# Supplementary material for: Finding a Needle in a Haystack: Distinguishing Mexican Maize Landraces Using a Small Number of SNPs
Source: Front Genet. 2017 Apr 18;8:45. doi: 10.3389/fgene.2017.00045 (PMC5394175; doi:10.3389/fgene.2017.00045)
Supplement: Supplementary file 1 [file Table1.PDF]

Supplementary Table 1. Sample information. Samples with name starting with number were obtained from CIMMYT, samples with names starting with ”maiz” were obtained from Arteaga et al. [2016].

Sample	Landrace	Sampling year	Longitude	Latitude	Altitude
14_2166	Comiteco	1946	-92.48	15.13	100
8_805	Comiteco	1961	-93.75	16.45	2241
9_1058	Comiteco	1961	-91.93	16.13	1400
17_10037	Comiteco	1952	-90.63	14.55	1943
37_30052	Comiteco	2010	-92.50	16.80	2008
32_26872	Comiteco	1972	-92.04	16.91	900
33_29890	Comiteco	2010	-92.71	16.76	1510
35_29971	Comiteco	2010	-92.50	15.58	1955
36_30008	Comiteco	2010	-92.72	16.76	1631
maiz_82	Comiteco	2009	-91.98	16.20	1553
maiz_80	Comiteco	2009	-93.10	16.62	455
maiz_150	Comiteco	2009	-92.02	16.25	1550
maiz_186	Comiteco	2009	-91.97	16.23	1550
maiz_183	Comiteco	2009	-91.94	16.20	1550
1_33	Conejo	1946	-100.70	18.30	210
2_44	Conejo	1947	-100.43	19.01	480
3_68	Conejo	1960	-101.33	17.70	180
5_168	Conejo	1952	-103.66	18.68	250
maiz_21	Conejo	2008	-98.74	17.78	1312
maiz_23	Conejo	2008	-98.74	17.78	1685
maiz_152	Conejo	2008	-98.68	17.89	1493
maiz_182	Conejo	2008	-98.66	17.75	1402
13_1861	Tehua	1952	-91.42	14.61	792
20_13297	Tehua	1972	-93.10	16.96	820
6_777	Tehua	1944	-92.71	15.87	1400
12_1849	Tehua	1953	-89.67	15.40	122
31_25784	Tehua	1972	-92.63	17.20	730
30_23861	Tehua	1947	-92.05	16.12	1604
maiz_124	Tehua	2009	-93.21	17.20	1545
maiz_125	Tehua	2009	-93.18	17.22	1525
7_802	Zapalote Chico	1961	-92.66	15.33	100
19_10473	Zapalote Chico	1973	-93.89	16.23	100
15_2270	Zapalote Chico	1946	-94.62	16.57	100
22_23130	Zapalote Chico	1998	-95.81	16.45	681
23_23142	Zapalote Chico	1998	-95.20	16.58	255
18_10463	Zapalote Chico	1972	-92.10	15.70	700
26_23547	Zapalote Chico	1999	-94.18	16.28	8
28_23597	Zapalote Chico	1999	-95.39	16.09	5
34_29952	Zapalote Chico	2009	-93.80	16.67	573
29_25029	Zapalote Chico	1976	-95.00	16.20	50
maiz_142	Zapalote Chico	2009	-93.00	16.36	654
21_16210	Zapalote Grande	1972	-93.77	16.08	41
4_137	Zapalote Grande	1958	-96.47	16.94	1000
16_2601	Zapalote Grande	1946	-93.79	16.10	31
10_1222	Zapalote Grande	1985	-	-	-
11_1773	Zapalote Grande	1946	-92.62	15.28	100
24_25020	Zapalote Grande	1976	-94.18	16.28	50
25_23217	Zapalote Grande	1962	-95.45	16.45	820
27_23552	Zapalote Grande	1999	-94.18	16.28	8
maiz_144	Zapalote Grande	2009	-93.00	16.36	654
