# Supplementary material for: Finding a Needle in a Haystack: Distinguishing Mexican Maize Landraces Using a Small Number of SNPs
Source: Front Genet. 2017 Apr 18;8:45. doi: 10.3389/fgene.2017.00045 (PMC5394175; doi:10.3389/fgene.2017.00045)
Supplement: Supplementary file 2 [file Table2.PDF]

Supplementary Table 2. Landrace informative SNPs.

	SNP	Chromosome	Coordinate	Gene	Annotation	DF
1	PZB01114.1	9	64,695,530	-	-	1
2	PZE-101080378	1	65,092,399	GRMZM2G042683	Uncharacterized	1
3	PZE-106049782	6	99,291,757	GRMZM2G153666	Polygalacturonase	1
4	PZE-109036638	9	50,198,296	-	-	1
5	PZE-109036749	9	50,383,446	-	-	1
6	PZE-109036755*	9	50,385,286	-	-	1
7	PZE-109037166*	9	51,669,851	-	-	1
8	SYN15460*	9	52,052,243	GRMZM2G100103	Uncharacterized	1
9	SYN15462*	9	52,052,297	GRMZM2G100103	Uncharacterized	1
10	PZE-109037353*	9	52,059,708	-	-	1
11	PZE-109039021*	9	59,462,243	GRMZM2G173090	Heat shock factor protein HSF30	1
12	PHM10525.11	8	124,752,481	-	-	2
13	PZE0006576497	2	50,310,642	-	-	2
14	PZE-100002058	2	49,681,375	-	-	2
15	PZE-101239396	1	286,726,652	GRMZM2G061084	Uncharacterized	2
16	PZE-104021070	4	22,553,514	-	-	2
17	PZE-108004274	8	4,285,891	GRMZM2G171122	Uncharacterized	2
18	PZE-108072700	8	125,607,200	GRMZM2G380515	Transcription Factor IIIA	2
19	PZE-108072703	8	125,610,778	GRMZM2G380515	Transcription Factor IIIA	2
20	PZE-109094812	9	141,330,945	-	-	2

* SNPs shared between the landrace and altitude informative SNPs.
