# Supplementary material for: Finding a Needle in a Haystack: Distinguishing Mexican Maize Landraces Using a Small Number of SNPs
Source: Front Genet. 2017 Apr 18;8:45. doi: 10.3389/fgene.2017.00045 (PMC5394175; doi:10.3389/fgene.2017.00045)
Supplement: Supplementary file 3 [file Table3.PDF]

Supplementary Table 3. Altitude informative SNPs.

	SNP	Chromosome	Coordinate	Gene	Annotation
1	PZE-102099083	2	116,801,868	-	-
2	PZE-102150323	2	197,663,601	GRMZM2G042741	SAUR40 Auxin-responsive
3	SYN25763	2	197,663,065	GRMZM2G042741	SAUR40 Auxin-responsive
4	PZE-102099063	2	116,694,897	GRMZM2G071744	Uncharacterized
5	PZE-102099223	2	117,348,457	-	-
6	PZE-104009802	4	6,691,056	GRMZM2G334631	Uncharacterized
7	PZE-105062945	5	62,229,361	GRMZM2G152111	Transformer-2 protein
8	PZE-105062976	5	62,231,214	-	-
9	PZE-109036755*	9	50,385,286	-	-
10	PZE-109037166*	9	51,669,851	-	-
11	SYN15460*	9	52,052,243	GRMZM2G100103	Uncharacterized
12	6SYN15462*	9	52,052,297	GRMZM2G100103	Uncharacterized
13	PZE-109037353*	9	52,059,708	-	-
14	PZE-109039002*	9	59,461,144	GRMZM2G173090	Heat shock factor protein HSF30

* SNPs shared between the landrace and altitude informative SNPs.
