# Supplementary material for: Finding a Needle in a Haystack: Distinguishing Mexican Maize Landraces Using a Small Number of SNPs
Source: Front Genet. 2017 Apr 18;8:45. doi: 10.3389/fgene.2017.00045 (PMC5394175; doi:10.3389/fgene.2017.00045)
Supplement: Supplementary file 4 [file Image1.PDF]

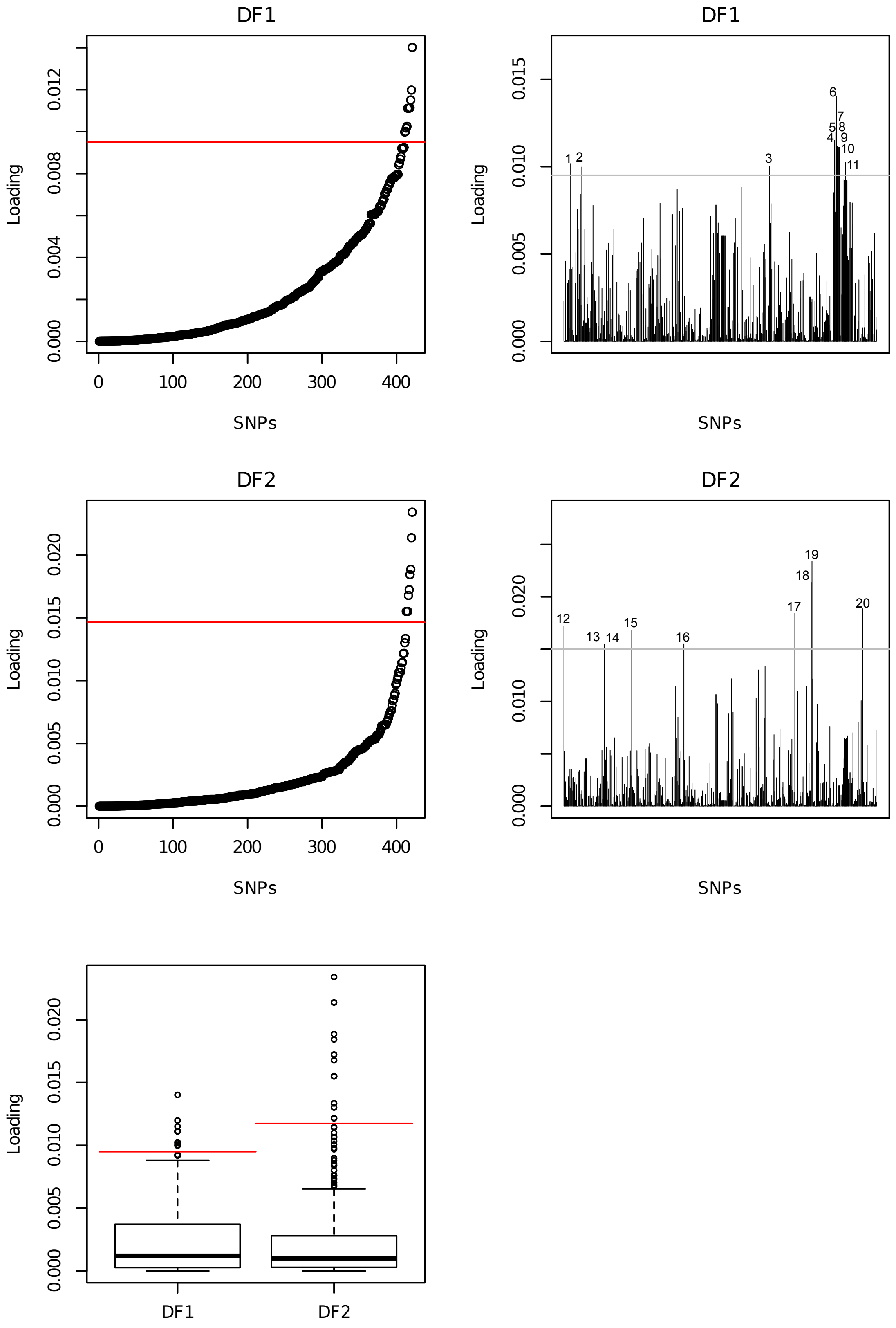

Supplementary Figure 1. SNP loading distribution for the two discriminant functions of the landrace DAPC model. Horizontal lines correspond to the loading cut-off for considering informative SNPs.The numbers correspond to the SNPs listed in Supplementary Table 2.
