# Supplementary material for: Finding a Needle in a Haystack: Distinguishing Mexican Maize Landraces Using a Small Number of SNPs
Source: Front Genet. 2017 Apr 18;8:45. doi: 10.3389/fgene.2017.00045 (PMC5394175; doi:10.3389/fgene.2017.00045)
Supplement: Supplementary file 5 [file Image2.PDF]

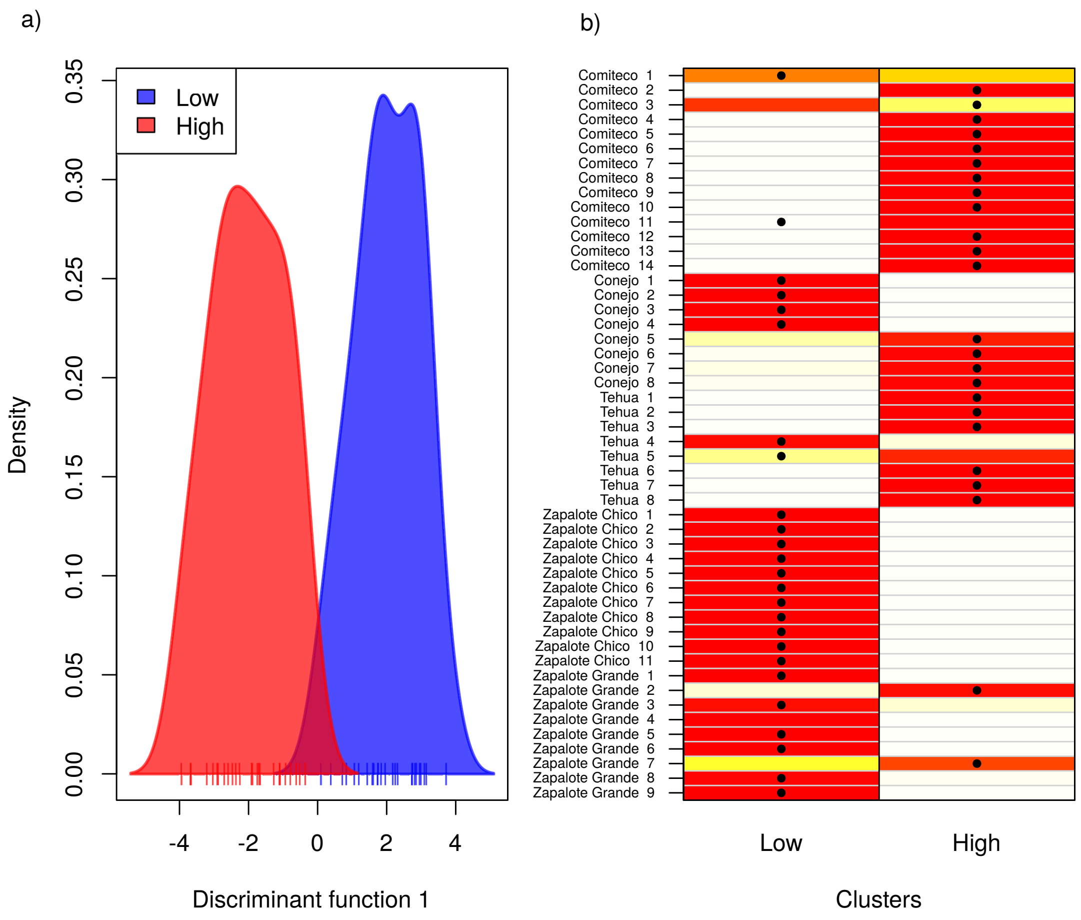

Supplementary Figure 2. Altitude DAPC. a) Classification plot: discriminant function for assigning each sample to an altitude category. b) Assignment plot: this plot shows the accuracy of the model to recover samples altitude, the color of each cell reflects the probability of assigning a given sample (row) to the corresponding altitude (column) with red colors meaning higher probability of assignment. The black dots in cells show the altitude of each sample.
