# Supplementary material for: Finding a Needle in a Haystack: Distinguishing Mexican Maize Landraces Using a Small Number of SNPs
Source: Front Genet. 2017 Apr 18;8:45. doi: 10.3389/fgene.2017.00045 (PMC5394175; doi:10.3389/fgene.2017.00045)
Supplement: Supplementary file 6 [file Image3.PDF]

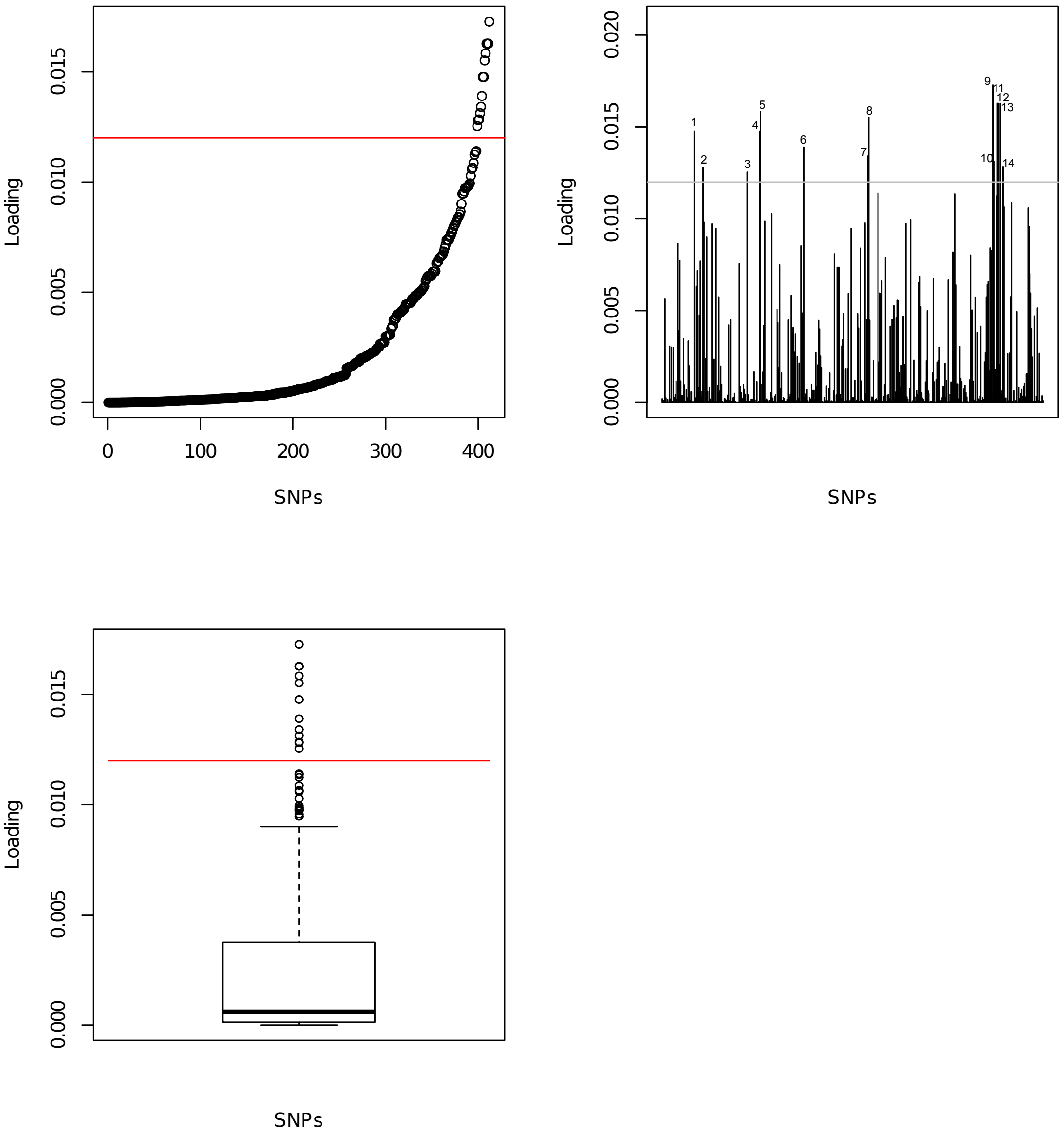

Supplementary Figure 3. SNP loading distribution for the discriminant function of the altitude DAPC model. Horizontal lines correspond to the loading cut-off for considering informative SNPs. The numbers correspond to the SNPs listed in Supplementary Table 3.
